# Cohesion and Adhesion Performance of Tannin-Glyoxal Adhesives at Different Formulations and Hardener Types for Bonding Particleboard Made of Areca (*Areca catechu*) Leaf Sheath

**DOI:** 10.3390/polym15163425

**Published:** 2023-08-16

**Authors:** Awanda Wira Anggini, Muhammad Adly Rahandi Lubis, Rita Kartika Sari, Antonios N. Papadopoulos, Petar Antov, Apri Heri Iswanto, Seng Hua Lee, Efri Mardawati, Lubos Kristak, Ika Juliana

**Affiliations:** 1 Department of Forest Products, Faculty of Forestry and Environment, IPB University, Bogor 16680, Indonesia; awandawira@gmail.com; 2 Research Center for Biomass and Bioproducts, National Research and Innovation Agency, Cibinong 16911, Indonesia; 3 Research Collaboration Center for Biomass and Biorefinery, BRIN and Universitas Padjadjaran, Jatinangor 45363, Indonesia; efri.mardawati@unpad.ac.id; 4 Laboratory of Wood Chemistry and Technology, Department of Forestry and Natural Environment, International Hellenic University, GR-661 00 Drama, Greece; antpap@for.ihu.gr; 5 Faculty of Forest Industry, University of Forestry, 1797 Sofia, Bulgaria; p.antov@ltu.bg; 6 Department of Forest Products Technology, Faculty of Forestry, Universitas Sumatera Utara, Kwala Bekala Campus, Medan 20355, Indonesia; apri@usu.ac.id; 7 Department of Wood Industry, Faculty of Applied Sciences, Universiti Teknologi MARA Pahang Branch Jengka Campus, Bandar Tun Razak 40450, Malaysia; leesenghua@hotmail.com; 8 Department of Agro-Industrial Technology, Universitas Padjadjaran, Jatinangor 45363, Indonesia; 9 Faculty of Wood Sciences and Technology, Technical University in Zvolen, 96001 Zvolen, Slovakia; kristak@tuzvo.sk; 10 PT. Greenie Alam Indonesia, Serpong, South Tangerang 15310, Indonesia; ikajuliana@greenie.id

**Keywords:** adhesive strength, areca particleboard, bio-based adhesives, cohesive strength, glyoxalated tannin

## Abstract

The use of alternative raw materials, such as agricultural biomass and by-products, in particleboard (PB) production is a viable approach to address the growing global demand for sustainable wood-based materials. The purpose of this study was to investigate the effect of the type of hardener and tannin-glyoxal (TG) adhesive formulation on the cohesion and adhesion performance of TG adhesives for areca-based PB. Two types of hardeners were used, NH_4_Cl and NaOH, and three adhesive formulations with tannin:glyoxal ratios (i.e., F1 (1:2), F2 (1:1), and F3 (2:1)) were applied to improve the cohesion performance and adhesion for areca-based TG adhesive for PB. The basic, chemical, and mechanical properties of the TG adhesive were investigated using a Fourier transform infrared spectrometer, rotational rheometer, dynamic mechanical analyzer (DMA), and X-ray diffractometer. The results show that a high glyoxal percentage increases the percentage of crystallinity in the adhesive. This shows that the increase in glyoxal is able to form better polymer bonds. DMA analysis shows that the adhesive is elastic and the use of NH_4_Cl hardener has better mechanical properties in thermodynamic changes than the adhesive using NaOH hardener. Finally, the adhesion performance of the TG adhesives on various types of hardeners and adhesive formulations was evaluated on areca-based PB panels. Regardless of the type of hardener, the TG adhesive made with F1 had better cohesion and adhesion properties compared to F2 and F3. Combining F1 with NH_4_Cl produced areca-based PB panels with better physical and mechanical qualities than the adhesive formulations F2 and F3, and complied with Type 8 particleboard according to SNI 03-2105-2006 standard.

## 1. Introduction

Particleboard is utilized in a variety of applications, accounting for 66% of all applications in the furniture sector [1] and 27% of all applications in civil construction for indoor use [2]. Globally, 96 million m^3^ of particleboard were manufactured in 2020, representing a 24.67% increase over the volume produced in 2010 [3]. Asia produces approximately 45–47% of particleboard, Europe produces 27–29%, and America produces 25–26% [4]. In manufacturing technology and wood bonding, advancements aid the evolution of composite products as a substitute for solid wood. Particleboard can be manufactured from various lignocellulosic materials derived from different plant wastes and by-products in particle form, bonded together with a polymeric adhesive. According to Nurdin [5], the particleboards fabricated from areca (*Areca catechu*) particles and tapioca-based adhesive exhibited satisfactory mechanical properties to fulfil the standard requirements. 

*Areca catechu* is also known as the areca palm, the areca nut palm, and the betel palm. This palm tree is known as Pinang in Indonesia. The large leaves of the mature areca have a thick sheath at the base that encircles the stem and protects the developing inflorescences until a few days before blooming. The leaves are massive, averaging 0.9–1.5 m long with 29 pinnae [6]. Many investigations have been conducted on the fibers extracted from areca leaves in order to optimize their utilization in value-added applications. TGA (thermogravimetric analysis), DSC (differential scanning calorimetry), and SEM (scanning electron microscopy) are some of the most widely used methods of analysis to evaluate the mechanical and thermal properties of short areca nut leaf sheath fiber reinforced polypropylene composites [7]. Areca can be utilized in the production of bio-based composite materials such as particleboard and fiberboard. Due to the significant depletion of forest resources, the effective valorization of non-wood lignocellulosic feedstocks and agricultural by-products in the development of sustainable, bio-based composites has gained increased interest from both industry and academia. 

The reconstituted wood-processing industry stands out with the problem of lowering the harmful formaldehyde emissions from wood-based panels such as PB. Approximately 95% of PB is made using formaldehyde-based synthetic adhesives, with urea-formaldehyde (UF) resin being the most extensively used type of adhesive, with an estimated total usage of approximately 11 million tons worldwide [8,9]. Of all formaldehyde-based wood adhesives, those containing urea can be more easily hydrolyzed and release free formaldehyde, which is associated with adverse human health effects, including cancer. Even at room temperature, urea’s reaction with formaldehyde produces unstable methylol groups that can be hydrolyzed by moisture [10]. The adoption of stricter standard requirements on formaldehyde from wood-based panels in Europe, the United States, Japan, and other countries, has led to the development of novel UF adhesives with low or very low formaldehyde content to produce wood-based panels with low formaldehyde emission levels [11,12]. Many academics are working to reduce the formaldehyde emissions of wood-based panels [13], such as the use of phenol-formaldehyde as a low formaldehyde emission adhesive for particle board, melamine-urea formaldehyde adhesive for particle board manufacture, and the addition of amines to urea formaldehyde adhesive to reduce formaldehyde emissions [14,15,16]. However, most of these technologies are associated with higher production costs or cause secondary pollutants.

Although formaldehyde has good adhesive capabilities, volatile organic molecules are hazardous [17]. Due to the increased public concern for lowering formaldehyde emissions, there is an increased industrial interest in using formaldehyde-free adhesives, such as poly (vinyl acetate), pMDI, and various bio-based adhesives. Consequently, various studies have been conducted with novel adhesive systems based on acetaldehyde, propionaldehyde, n-butyraldehyde, or furfural. Glyoxal was also used as a formaldehyde substitute to prepare tannin/furanic stiff foams. In pine foams, glyoxal can replace formaldehyde [18]. Dialdehyde glyoxal is an environmentally friendly alternative to formaldehyde for the reaction of lignin. It is a naturally occurring chemical produced as a byproduct of biological processes and lipid oxidation [19]. In biomass-based adhesives, glyoxal was used instead of formaldehyde, and the plywood produced had better mechanical qualities [20]. Glyoxal has a low vapor pressure and toxicity and is reactive due to its two neighboring carbonyl groups [19]. Glyoxal shows similar reactions to formaldehyde with phenolic rings and has thus been used as a formaldehyde substitute in wood adhesives [19,21,22]. The glyoxalation of lignin and glyoxal curing of tannin have already been suggested as viable ways for producing adhesives [23].

Tannins, the second most abundant source of polyphenols in biomass after lignin, are appealing components for producing green chemicals and polymers. Tannins have a higher reactivity than phenols. Tannins can act as continuous reinforcers by creating a large number of hydrogen bonds with proteins or other oxygen-containing molecules [24]. Tannins and other natural components can be employed in particleboard adhesives [25,26,27]. A study demonstrated the practical viability of replacing standard urea–formaldehyde systems with cornstarch–mimosa tannin–urea formaldehyde resins in classic adhesive compositions [28]. The use of tannin extract as the main ingredient for adhesives is still relatively small because condensed tannin extracts tend to have brittle and weak bonds, so the tannin extract is mixed with other commercial adhesives such as phenol formaldehyde or urea formaldehyde [29,30]. In the research of Siswanto for the manufacture of tannin-urea-formaldehyde (TUF) adhesive, they used tannin extract in the process of making particle board and there is a Santoso patent which uses mangium liquid extract as an adhesive for wood products [31,32].

Mangium (*Acacia mangium* Willd.) is a type of tree commonly used as a raw material for the pulp and paper industry. Around 52.63% of log production in Indonesia in 2020 (61.02 million m^3^) was dominated by mangium [33]. The bark waste is predicted to be around ±10% for each log. This waste is still underutilized and mainly used for producing heat. Mangium bark contains a high concentration of tannin compounds and can be used as a bio-based feedstock for producing wood adhesives [34,35]. The extraction of mangium bark with hot water yields a tannin extract with a tannin concentration of 44.73–54.09% [36]. 

This study aimed to investigate the effect of a tannin-glyoxal adhesive hardener on the adhesion and cohesion performance of particleboard manufactured from areca leaf sheaths. To improve particleboard adhesion and cohesion performance, NH_4_Cl (acidic conditions) and NaOH (alkaline conditions) were utilized as adhesive hardeners. In this work, the basic, chemical, and mechanical properties of the developed tannin-glyoxal adhesives were investigated and the effect of hardener types was evaluated by using several techniques, such as Fourier transform infrared (FTIR) spectroscopy, rheological properties, dynamic mechanical analysis (DMA), and X-ray diffractometer (XRD). Finally, the adhesion and cohesion performance of the particleboard panels, bonded with tannin-glyoxal adhesives at different hardener levels was evaluated by comparison with a commercial UF resin.

## 2. Materials and Methods

### 2.1. Materials

Mangium bark (*A. mangium*) supplied by Tanjung Enim Lestari Pulp and Paper (Palembang, Indonesia); Aquades, Glyoxal (40%, analytical grade, Merck, Martillac, France), aluminum foil (Klinpak, Jakarta, Indonesia); Whatman filter papers (Whatman, Buckinghamshire, United Kingdom); NH_4_Cl 20% and NaOH 20% were used to perform the experiments. In the manufacture of particle board, the adhesive is added with 1% paraffin wax which is mixed with the adhesive. Areca leaf sheath particles were obtained from PT Greenie Alam, Indonesia (Tangerang, Indonesia). 

The tools used in this study were magnetic stirrers, calipers, digital pH meters, rotary evaporators (Rotavaapor, Buchi R–300, Muttenz, Switzerland), rotational rheometer (RheolabQC, AntonPaar, Graz, Austria), Erlenmeyer, Fourier transform infrared spectroscopy (FTIR, SpectrumTwo, Perkin Elmer, Waltham, MA, USA), dynamic mechanical analyzer (DMA 8000, PerkinElmer, Waltham, MA, USA), oven (Memmert celsius 10.0, Memmert, Germany), universal testing machine (UTM, 10 kN, Shimadzu, Kyoto, Japan), hot pressing, pyrolysis gas chromatography mass spectrometry (Py-GCMS) (Shimadzu, Kyoto, Japan), oven (Memmert celsius 10.0, Memmert, Germany), and desiccator.

### 2.2. Preparation of Tannin Bark Extract

**Figure 1:**
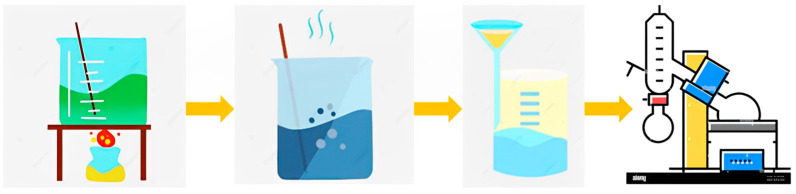
Extraction of tannins from mangium bark.

Mangium bark was powdered using a dish mill and hammer mill in the early stages of preparation, with a 40-mesh size filter retained at 60 mesh; then, tannins were produced through the maceration extraction method. Tannins were extracted via the hot water extraction method [37]. About 100 g of mangium bark powder and 1000 mL of distilled water (*w*/*v* 1:10) were mixed into a glass jar, heated at 60 °C for 6 h, and left for 24 h at room temperature (Figure 1). The extract obtained was separated from the powder through a filter. The extraction was repeated with the same ratio until the filtrate was clear. After receiving the filtrate, the filtrate was evaporated in a rotary evaporator at 60 °C and 22 mBar pressure to obtain a viscous extract. 

The percentage of tannin yield was estimated by measuring the dry powder produced by the freeze drier and adjusting for moisture content (MC) using the equation:

$$\%\mathrm{Yield}\;\mathrm{of}\;\mathrm{Tannin}\;=\frac{\mathrm{weight}\;\mathrm{of}\;\mathrm{extract}\;\mathrm{tannin}\;\left(g\right)}{\mathrm{weight}\;\mathrm{of}\;\mathrm{dried}\;\mathrm{oven}\;\mathrm{bark}\;\left(g\right)}\times 100\%$$

To determine the type of tannins obtained, qualitative tannin testing was carried out. As much as 5 g of tannin extract was dissolved in 50 mL of distilled water and then heated to boiling for 30 min while stirring. It was allowed to stand a few minutes, precipitated, then filtered with filter paper. The filtrate was put into a 250 mL volumetric flask. This procedure was repeated until the solution reacted with the FeCl_3_ solution.

Three metrics were used to characterize the tannin extract: solids content, functional group analysis, and the Stiasny number. The total tannin solids in the condensed tannin extract are expressed as a percentage of solids content. One gram of the test sample was placed on aluminum foil and placed in an oven at 103 °C for 3 h to allow the extract’s solution to evaporate entirely. The aluminum foil was removed and weighed after being placed in a desiccator. The formula used for calculating the solids content was:

$$\mathrm{Solids}\;\mathrm{content}\;(\%)=\frac{\mathrm{BA}\;-\;\mathrm{BK}}{\mathrm{BB}\;-\;\mathrm{BK}}\times 100\%$$

where:

BA is the weight of aluminum foil with sample oven-dried (g), 

BB is the weight of aluminum foil with sample before oven-dried (g), 

BK is the weight aluminum foil (g).

The polyphenolic content of the extract was determined using the Stiasny number reaction. A 150 mL flask was pipetted with 50 mL (0.4% *w*/*w*) of tannin solution. The mixture was then heated for 30 min with an aqueous solution of formaldehyde (37%; 5 mL) and hydrochloric acid (10 M; 5 mL). The reaction mixture was filtered while still hot, and the residue was dried to a constant weight in an oven at 105 °C. The Stiasny number is the percentage of the weight of the oven-dried residue to the tannin extract’s total dissolved solids content.

$$\mathrm{Stiasny}\;\mathrm{number}\;(\%)=\frac{\mathrm{the}\;\mathrm{weight}\;\mathrm{of}\;\mathrm{the}\;\mathrm{remaining}\;\mathrm{dry}\;\mathrm{solids}}{\;\mathrm{dry}\;\mathrm{extract}\;\tan\mathrm{nin}\;\mathrm{weight}}\times 100\%$$

### 2.3. Manufacture of TG Adhesive

**Figure 2:**
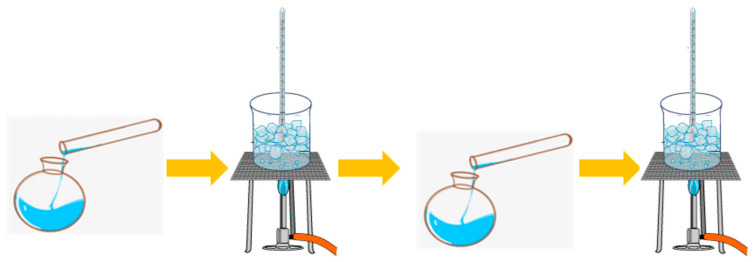
TG adhesive manufacturing process.

The pH of a viscous tannin solution (solids content 91%) was modified with 20% NaOH until alkaline. After that, the temperature was increased to 80 °C, the glyoxal solution was slowly added using a dropper, and the temperature was maintained for one h to produce TG adhesive (Figure 2). The adhesive that was made was indicated by the color of the adhesive being reddish black and thicker than the tannins and glyoxal before making the adhesive. Table 1 illustrates the formulation ratios.

### 2.4. Characterization of TG Adhesive

A rotational rheometer was used to investigate the flow behavior of the TG adhesives. Viscosity, cohesion strength, and relaxation modulus were measured at 25 and 80 °C with a dynamic shear rate of 1–500/s.

The gelatinization time test was performed by placing 50 g of the test material in a glass, then heating it to a temperature of 135 ± 2 °C, observing the gelatinization using a gelation timer, and recording the time. A gelation time meter was used to measure the gelation time of the TG adhesives in boiling water.

FTIR spectroscopy was combined with universal attenuated total reflectance (UATR) to evaluate the functional groups of liquid and solid adhesives. Samples of 1 g were prepared to investigate the chemical differences between adhesives with TG ratios of F1, F2, and F3. The mixture was subsequently placed in a petri dish and dried for 24 h in a vacuum oven set to 50 °C. After that, all samples were pulverized into powder for examination. The KBr pellet technique (5 mg sample in 300 mg KBr) was used to analyze liquid and powder adhesive samples with a resolution of 2 cm^−1^ and 16 scans per sample over 400–4000 cm^−1^. To normalize the adhesive spectrum, min–max normalization was used.

DMA sampling was carried out. Whatman filter papers with a size (50 × 10 × 0.2) mm^3^ were impregnated with an adhesive solution at a 1 mg/mm^3^ resin-loading ratio. Then, the paper was pre-cured at 50 °C for 30 min. The prepared specimens were set up in the DMA’s double cantilever mode. For dynamic DMA scanning, the temperature was increased from room temperature to 300 °C with a heating rate of 5 °C/min, a frequency of 1 Hz, and a strain rate of 0.005%. 

XRD was used to assess adhesive crystallinity. Each sample’s XRD patterns were acquired by scanning at room temperature with an angle range of 10–80° and a rate of 0.02°/min.

Around 500–600 g of adhesive sample was placed in the eco-cup SF PYI-EC50F and coated with glass fiber before being analyzed with Py-GCMS. The eco-cup had been pyrolyzed for 0.1 min at 500 °C, utilizing a multi-shot pyrolysis connected to a Shimadzu QP-2020 NX GC/MS system equipped with an SH-Rxi-column. The film thickness of 5Sil MS was (30 × 0.25 × 0.25) mm^3^ electron energy of 70 eV, and helium as a carrier gas. A pressure of 20.0 kPa was utilized (15.9 mL/min, column flow 0.61 mL/min). The temperature profile for GC begins at 50 °C and is held for 1 min before increasing to 280 °C at the rate of 5 °C per minute for 13 min. The pyrolysis products were found by comparing retention time and mass spectrum data from the 2017 NIST LIBRARY program.

### 2.5. Particleboard Preparation and Its Properties

**Figure 3:**
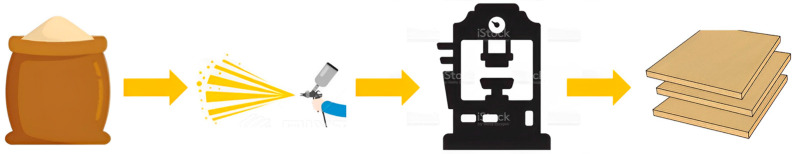
Areca particleboard manufacturing process.

The areca leaf sheath was milled using a hammer mill to obtain particles. The particles were dried until reaching a moisture content of 5.6%. The proportion of particle size was observed using 14–16 mesh. Particleboard was manufactured by mixing adhesive, 3% hardener, 1% wax, and particles, with the adhesive amount being 10% of the dry particle weight (Table 2). Boards measuring (25 × 25 × 1.2) cm^3^ were fabricated with varying hardener types. The compression temperature used was 150 °C, and the duration of hot press was 15 min. The target density and compression pressure were 0.80 g/cm^3^ and 10 MPa, respectively. Next, the boards were conditioned at room temperature for 14 days (Figure 3).

SNI 03-2105-2006 was used to test the board’s physical and mechanical properties [38]. Density, moisture content, thickness expansion, and water absorption were the physical properties examined, while the mechanical properties were modulus of elasticity (MOE), bending strength (MOR), and internal bond strength (IB).

Density testing was carried out using samples measuring (5 × 5 × 1.2) cm^3^ in length, width, and thickness. The determination of density was expressed in the results of the comparison between the weight and volume of the board. The density of the particleboard was calculated using an equation that refers to the SNI 03-2105-2006 standard.

$$\mathrm{Density}\;(g/{\mathrm{cm}}^{3})=\frac{M}{V}$$

where:

M is the weight of particleboard (g), and

V is the volume of particleboard (cm^3^).

A moisture content test was performed on samples measuring (5 × 5 × 1.2) cm^3^ in length, width, and thickness. The moisture content test was calculated using the difference between the initial weight (BB) and the final weight after 24 h of drying in the oven at 103 ± 2 °C. The water content value was calculated using an equation based on the SNI 03-2105-2006 standard.

$$\mathrm{Moisture}\;\mathrm{content}\;(\%)=\frac{\mathrm{BB}\;-\;\mathrm{BKT}}{\mathrm{BKT}}\times 100\%$$

where:

BB is the weight of the sample before drying (g), and

BKT is the weight of the sample after drying (g).

Water absorption tests were performed on samples measuring (5 × 5 × 1.2) cm^3^. The difference in weight before soaking (B1) and weight after immersion in water for 24 h (B2) was measured. The water absorption value was derived using a calculation based on the SNI 03-2105-2006 standard.

$$\mathrm{Water}\;\mathrm{absorption}\;(\%)=\frac{B2-\;B1}{B1}\times 100\%$$

where:

B1 is the weight of the sample before soaking (g),

B2 is the weight of the sample after soaking (g).

Thickness swelling tests were performed on samples of length, width, and thickness (5 × 5 × 1.2) cm^3^. Measurements were made by measuring the difference in initial thickness before soaking (T1) and after soaking for 24 h (T2). The thickness expansion value was calculated using an equation that refers to the SNI 03-2105-2006 standard.

$$\mathrm{Thickness}\;\mathrm{swelling}\;(\%)=\frac{T2-\;T1}{T1}\times 100$$

where:

T1 is the thickness of the sample before soaking (mm),

T2 is the thickness of the sample after soaking (mm).

The particleboard was cut into specimen cubes (20 × 20 × 15) mm^3^. The particleboard was hydrolyzed under two conditions (25 °C and 100 °C for 1 h) with three replicates for each sample. Then, the board was dried in the oven for 24 h at a temperature of 103 ± 2 °C. The mass loss, pH of the extract solution, and functional groups of the particleboard following hydrolysis were determined to compare particleboard recycling.

$$\mathrm{Weight}\;\mathrm{loss}\;(\%)=\frac{\left(\left(B_{1}-\;B\right)-{\;B}_{2}\right)}{\left(B_{1}-\;B\right)}\times 100$$

where:

B is the sample weight before hydrolysis (g),

B_1_ is the weight of the water contained in the board (g),

B_2_ is the sample weight after drying (g).

Modulus of elasticity (MOE) and Modulus of rigidity (MOR) tests were performed on samples measuring (20 × 5 × 1.2) cm^3^. The universal testing machine was then used to test the results. Flexural and fracture strength tests were performed at 10 mm/minute loading speeds. The MOE and MOR are determined using an equation based on the SNI 03-2105-2006 standard.

$$\mathrm{Modulus}\;\mathrm{of}\;\mathrm{elasticity}\;(\mathrm{kg}/{\mathrm{cm}}^{3})=\frac{∆{\mathrm{PL}}^{3}}{4∆{\mathrm{Ybh}}^{3}}\;\mathrm{Modulus}\;\mathrm{of}\;\mathrm{rigidity}\;(\mathrm{kg}/{\mathrm{cm}}^{3})=\frac{3P_{\max}L}{2{\mathrm{bh}}^{2}}$$

where:

P_max_ is the maximum load (N),

P is the load below the limit of proportion (N),

Y is the deflection at load P (mm),

L is the pacing distance (mm),

b is the width of the test sample (mm),

h is the thickness of the test sample (mm).

The test was carried out using samples of (5 × 5 × 1.2) cm^3^, which were then bonded to two iron blocks with epoxy glue and left to dry for 24 h. The two iron blocks were then dragged perpendicular to the surface of the sample using a universal testing machine at a loading speed of 2 mm/min to a maximum load. The adhesive strength was calculated based on the SNI 03-2105-2006 standard.

$$\mathrm{Internal}\;\mathrm{bonding}\;(\mathrm{IB})\;\mathrm{strength}\;(\mathrm{MPa})=\frac{P}{A}$$

where:

P is the maximum load (N),

A is the cross-sectional area (mm^2^).

### 2.6. Statistical Analysis

Data analysis was performed using the Microsoft Excel 2017 application and IMB SPSS Statistics 20. The test data for solids content and specific gravity were analyzed using compare means, whereas the viscosity and gelatinization time of TG adhesive, as well as the physical and mechanical properties of particleboard, were analyzed using completely randomized factorial design. Three replicates were tested for every treatment. Duncan’s new multiple range test was employed to further separate the mean values that showed the significant effect of the interaction of the two factors or of each factor at 95% confidence interval.

## 3. Results and Discussion

### 3.1. Characterization of Tannin Obtained from Acacia mangium Bark

The characteristics of the viscous tannin extract are presented in Table 3. The yield of the viscous tannin extract obtained using the hot water maceration method was 23.33% in this study. The difference in the yield value was due to the difference in the value of the moisture content (MC) of the mangium bark powder used. High MC materials complicate the extraction process and require more solvent than low MC materials. In addition, the MC of the material affects the shelf life and resistance to fungal attack. Raw materials with a high MC usually have a short shelf life. The amount of tannin extract included in the tannin viscous extract is indicated by the solids content of the tannin extract. The tannin extract produced in this study had a solids content of 91.36%. Based on qualitative tannin testing, the type of tannin obtained from hot water extraction of mangium bark is condensed tannin. This is indicated by the formation of a green-black precipitate after the addition of FeCl_3_. Gallotannins and ellagitannins will give a blue-black precipitate and condensed tannins will give a greenish-black precipitate [39].

**Figure 4:**
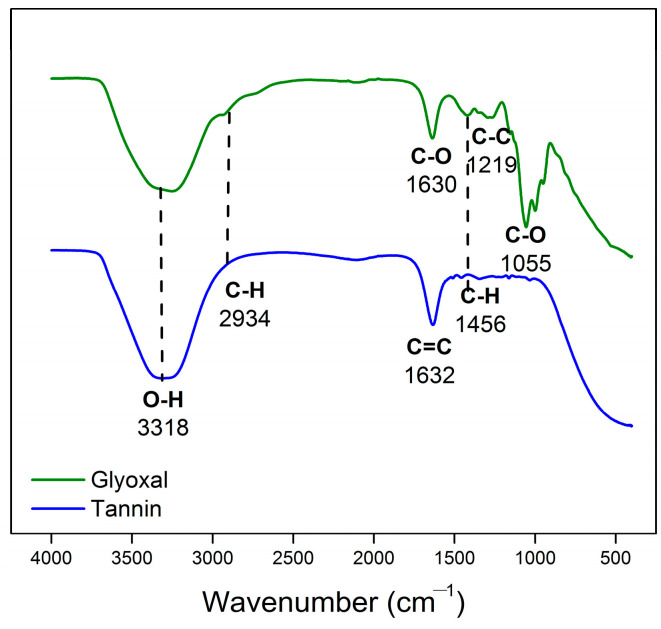
FTIR spectra of glyoxal and viscous tannin extract.

The level of reactivity of the phenolic compounds contained in mangium bark extract to formaldehyde is expressed by the Stiasny number. The Stiasny number indicates the reactivity of the tannin compound contained, and a number reaching one hundred (100) suggests that the tannin extract contains polyphenols which are very reactive in the polymerization process [42]. The Stiasny number of the tannin obtained in this study was 73.17%. These findings clearly show that this form of tannin has a substantially higher percentage of reactive tannins than the minimum requirement of 65% [41]. Before mixing, the tannin and glyoxal functional groups were analyzed; the results are depicted in Figure 4. At wave number 3318 cm^−1^, the tannin and glyoxal contained hydroxyl groups (–OH) with intermolecular bonds. At wave number 2934 cm^−1^, C-H bonds between glyoxal molecules were detected. At wave number 1632 cm^−1^, an aromatic C=C strain was observed in the tannins. Then, at wave number 1055 cm^−1^, there was a C-O bond strain on the glyoxal.

### 3.2. Physical Properties and Characteristics of TG Adhesive

The physical properties and characteristics of the tannin-glyoxal adhesives are summarized in Table 4. It can be observed that the solids content (%) and specific gravity decreased as the percentage of glyoxal decreased. In this study, adhesive F1 showed the highest percentage of solids content. This might be attributed to reaching the optimal level of glyoxal to react with tannins. Likewise, the viscosity value of the adhesive decreased with an increase in the percentage of tannin [43]. This shows that a higher percentage of tannin compared to glyoxal will cause some tannins to remain unreacted because the glyoxal will become the limiting reagent during the reaction, reducing the viscosity value. Acid hardeners can maintain viscosity values better than adhesives with alkaline hardeners.

Gelatination time measured at 135 °C shows the difference in how long it takes for the adhesive to gel. The adhesive with NaOH hardener required a longer gelatinization than the adhesive developed with NH_4_Cl hardener and without hardener. The longer the gelatination time, the longer the shelf life of the adhesive because it takes a long time to become a gel. In general, NH_4_Cl is considered a traditional catalyzing reaction that, when reacted with formaldehyde, will produce acid during the curing process, accelerating the condensation reaction and making the resin harden [44]. The analysis of variance showed that the type of formulation and hardener factor significantly affected the viscosity value and gelatination time.

**Figure 5:**
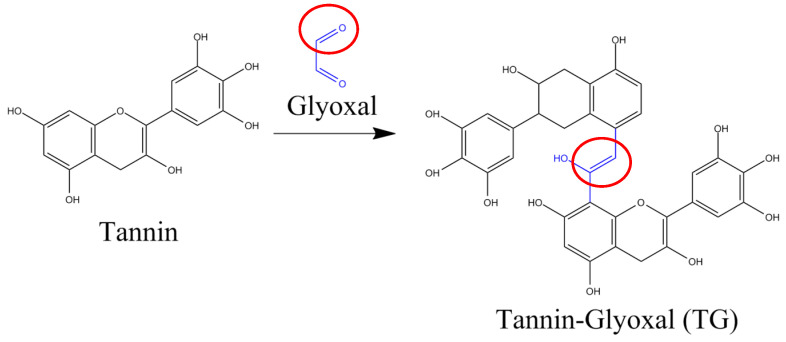
Functional group C=O in the reaction scheme of tannin and glyoxal adhesives.

**Figure 6:**
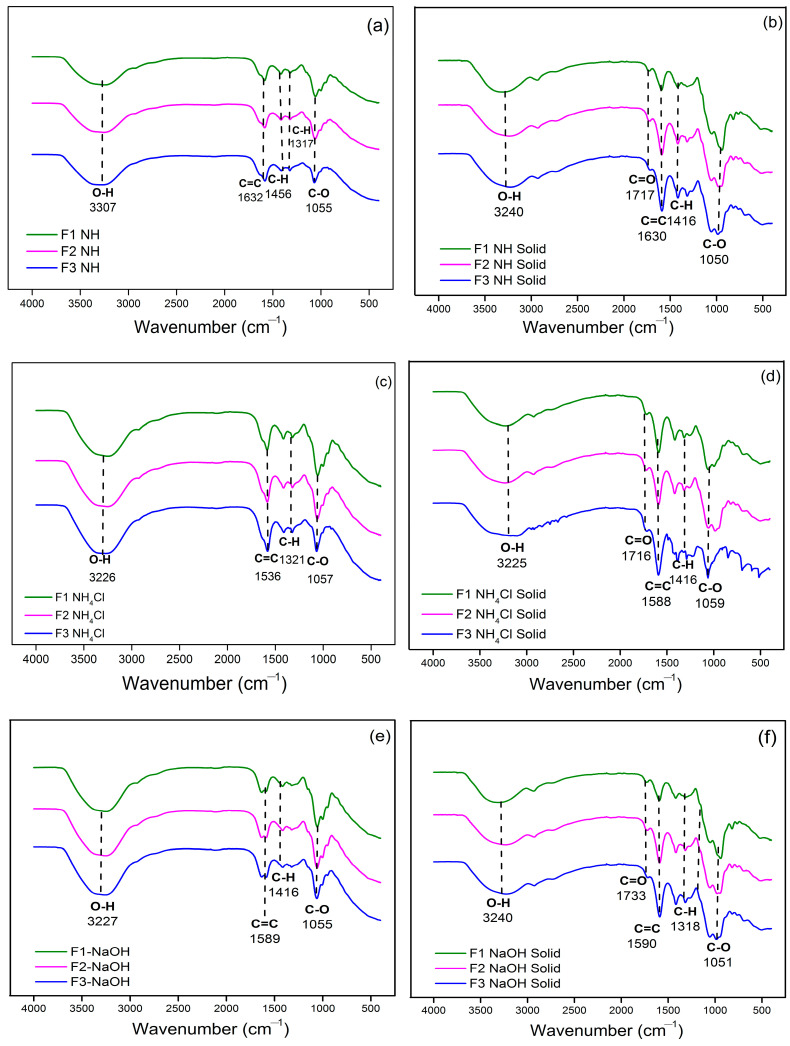
Spectrum of functional groups of liquid TG adhesive without hardener (**a**), solid TG adhesive without hardener (**b**), liquid TG adhesive with NH_4_Cl hardener (**c**), solid TG adhesive with NH_4_Cl hardener (**d**), liquid TG adhesive with NaOH hardener (**e**) and solid TG adhesive with NaOH hardener (**f**).

Confirmation of the functional groups of both liquid and solid tannin-glyoxal adhesives using FTIR produced the same relative absorption peaks as shown in Figure 5 and Figure 6a,b. Figure 5 explains the presence of a C=O group (red circle) at wave number 1717 cm^−1^ originating from glyoxal compounds. The absorption peaks at wave numbers 3325–3300 cm^−1^ suggested absorption from the vibrational strain of the phenolic –OH group [45], while the broad peak indicates the presence of created hydrogen bonds [46]. The occurrence of –OH absorption peaks may also be attributed to the water content of the tannin-glyoxal adhesive; because the solids content was 47%, a water content of 53% can be calculated. Furthermore, the absorption peak at 1416 cm^−1^ suggested that the aromatic components obtained from the tannin structure were stretching their C-H bonds. The peak of the absorption wave number 1059 cm^−1^ indicated the presence of the C-O bond strain originating from the glyoxal structure. Furthermore, a strong absorption peak at 1581 to 1589 cm^−1^ indicated a C=C stretching vibration of the aromatic ring. In contrast, these peaks were undetected in the glyoxal spectra. These results suggest that crosslinking between tannins and glyoxal was achieved. 

Figure 6c–f describes the analysis of the functional groups of the TG adhesive added with acid or base hardener. The addition of this hardener did not affect the functional groups formed from the adhesive. However, the dried adhesive had the C=O functional group at wave number 1716 cm^−1^ which comes from a glyoxal compound. After the adhesive was dried, the intensity of the functional groups detected became sharper.

**Figure 7:**
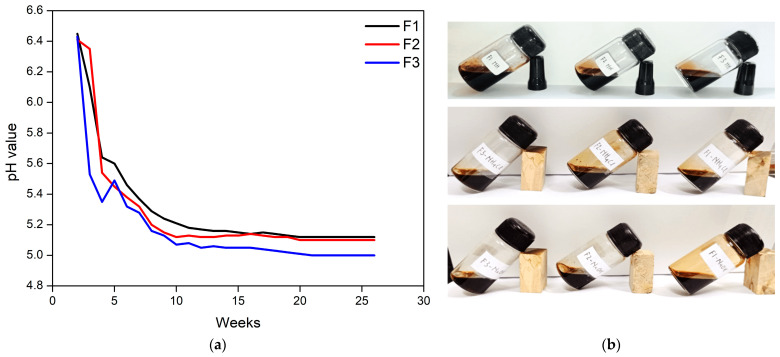
Adhesive storage time based on the pH value (**a**), and adhesive (**b**).

The results highlighted that the pH of the adhesive was alkaline and in liquid form at week 0, then dropped dramatically until the sixth week (Figure 7). It is suspected that there was a reaction between tannins and glyoxal after the adhesive synthesis process was completed [47]. Commercial adhesives have an alkaline pH because a high pH will slow down the curing process [48]. However, the tannin-glyoxal adhesive did not harden even when the pH of the adhesive became acidic. After the 6th week, the pH of the adhesive decreased slowly and tended to be stable; this indicated no reaction to the adhesive. The acidic nature of the tannin-glyoxal adhesive is caused by the constituent materials, namely tannin (pH 5) and glyoxal (pH 3.01), which are acidic.

**Figure 8:**
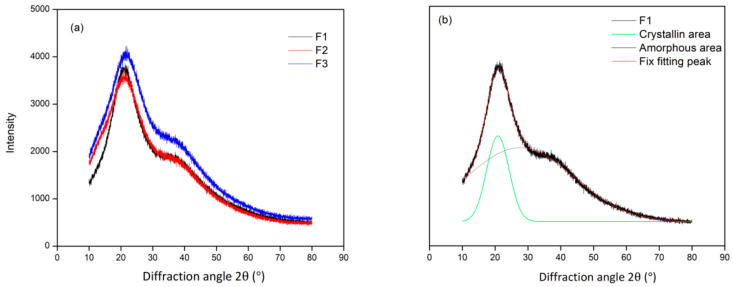
XRD diffractogram of TG at different formulas (**a**), and example of Gaussian function for deconvolution of XRD patterns of TG adhesive (**b**).

The XRD diffractogram revealed that the crystallinity of the F1 adhesive was greater than that of the F2 and F3 adhesives (Figure 8a). The crystallinity percentages of F1, F2, and F3 were 56.29, 50.10, and 40.22%, respectively. This result is directly proportional to the level of adhesive solids. The degree of crystallinity is calculated by dividing the total area of the crystalline and amorphous areas by the area of the crystalline region [49]. The results showed that the degree of crystallinity decreased as the percentage of glyoxal in the adhesive decreased. Glyoxal is known to be able to bind to the reactive side of tannin so that more glyoxal can bind only to one reactive side of tannin. The crystallinity level of TG adhesive was obtained by applying the Gaussian function to determine the crystallinity region of the XRD diffractogram (Figure 8b). Peak number 1 is designated as the crystalline region, while peak number 2 is associated with the amorphous region. This indicates that the crystal region in the three TG adhesive formulas occurred at 20.3°.

**Figure 9:**
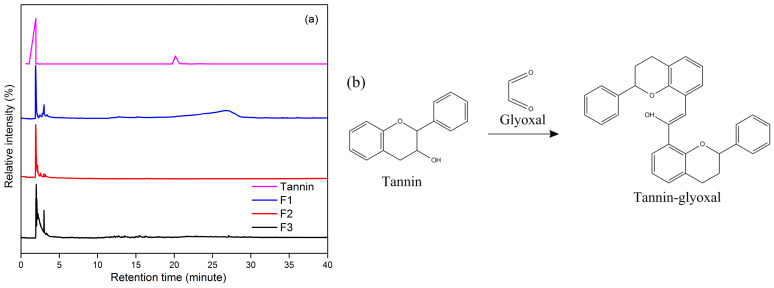
Py-GCMS analysis of TG adhesive (**a**), and reaction of tannin and glyoxal (**b**).

Based on the results of Py-GCMS analysis (Figure 9), the tannins exhibited a peak of the carbon dioxide (CO_2_) component with the highest relative abundance (%), which was 82.49% at a retention time of 1.931 min. After the tannin is reacted with glyoxal to form an adhesive, the carbon dioxide component also has the highest relative abundance compared to other compound components. Carbon dioxide is known to function as a continuous comonomer for the synthesis of polycarbonate, polyurea and polyurethane [50]. Furthermore, several component fractions resulting from tannin pyrolysis with a relatively low tannin abundance, such as nickel tetracarbonyl (0.87 and 0.22%), acetone (0.77), 2-(phenylmethyl) phenol, trimethylsilyl ether (0.77), 18%, and 2-Methyl-5-hydroxy benzofuran (0.48%), were present.

**Figure 10:**
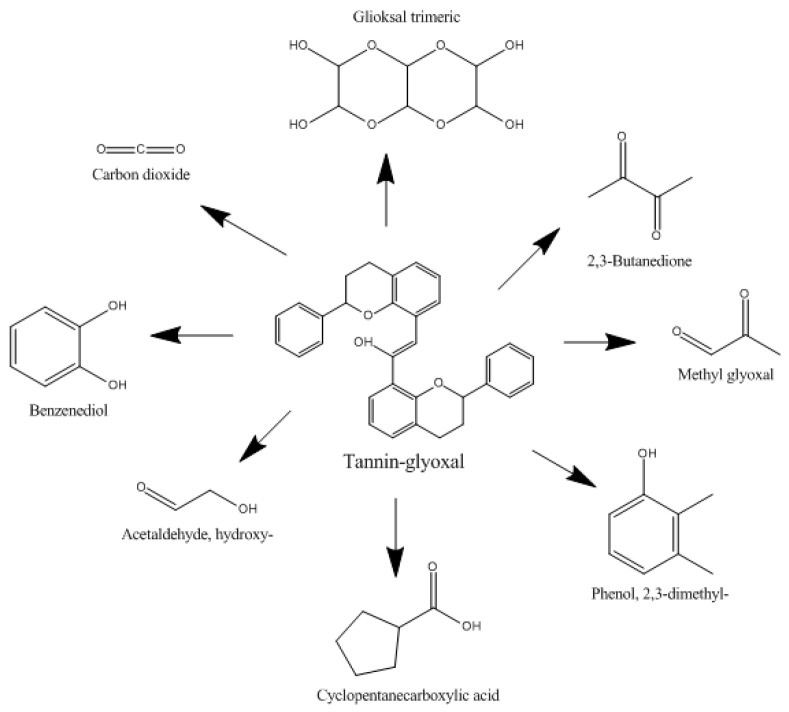
TG adhesive pyrolysis compound.

The tannin-glyoxal adhesive underwent a pyrolysis process and was degraded into certain molecular fractions, showing the retention time of 26.69 min in the F1 adhesive formula which indicated the presence of trimeric glyoxal with a relative abundance of 5.56% derived from glyoxal molecules. In the adhesive formula F2, a methyl glyoxal compound was detected at a retention time of 2.16 min with a relative abundance of 11.37% which also came from glyoxal molecules (Figure 10). The presence of benzene compounds was determined in the three tannin-glyoxal adhesive formulations. Tannins emit carbon dioxide and benzene triol at high temperatures. Benzene triol is a byproduct of the tannin structure’s decarboxylation of the outer gallic acid layer [51].

### 3.3. TG Adhesive Cohesion Properties

**Figure 11:**
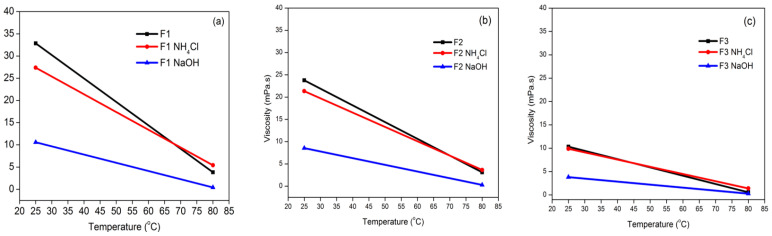
TG adhesive viscosity values of formulas F1 (**a**), F2 (**b**), and F3 (**c**) as a function of temperature.

The connection between shear stress (τ) and shear rate (γ) characterizes the rheological flow behavior of each fluid. The ratio of shear stress to shear rate is known as viscosity (η), which may also be regarded as a measure of resistance produced by neighboring layers during the flow of a liquid suspension [52]. The capacity of the adhesive to move from one surface to another on the glued wood to form a continuous layer and distribute evenly is referred to as adhesive viscosity. TG adhesives have the same trend in viscosity, i.e., the temperature increase is associated with decreased viscosity (Figure 11a–c). The decrease in dynamic viscosity occurs due to the broken polymer chain bonds due to high temperatures causing the polymer chains to become random so that the interactions between the molecular chains are reduced [53]. The F1 adhesive had a higher viscosity value than The F2 and F3 adhesives. This shows that a high percentage of glyoxal can increase the bonding of the polymer chains in the adhesive. Glyoxal is known to be able to increase cross-linking because glyoxal has a steric effect on the structure of cross-linked fiber networks [54]. A higher percentage of glyoxal will produce a polymer that has strong interchain forces. This was also confirmed based on the percentage of adhesive crystallinity, where a higher percentage of adhesive crystallinity indicates that the adhesive has a stronger polymer bond. This corresponds to the higher viscosity value of F1 compared to F2 and F3 because the adhesive is composed of regular polymer chains so that the viscosity value becomes higher.

**Figure 12:**
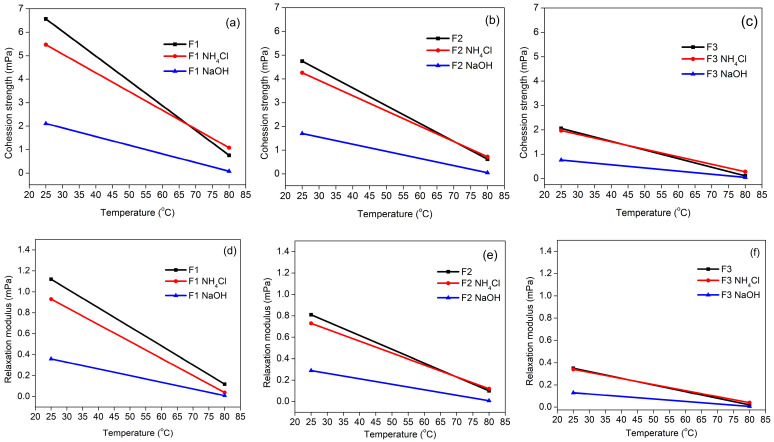
Cohesion strength values of F1 (**a**), F2 (**b**), F3 (**c**) and relaxation modulus of F1 (**d**), F2 (**e**), and F3 (**f**) of TG adhesives as a function of temperature.

The interaction and intermolecular forces of the adhesive will cause the cohesive strength to increase. The cohesive strength of TG showed the same trend as the viscosity. As the temperature increased, the cohesion strength decreased (Figure 12a–c). The F1 adhesive, both at 25 and 80 °C, had a higher cohesion strength value than the other adhesive formulas under the same conditions. Because it is used to quantify stress relaxation over time, the relaxation modulus is an important property of viscoelastic materials. The higher the temperature, the lower the value of the relaxation modulus (Figure 12d–f). With a greater reduction in the relaxation modulus, it can be concluded that the adhesive dissipates more energy with increasing temperature.

The thermo-mechanical characteristics of the TG adhesive were investigated via DMA testing. The viscoelastic properties of polymers are expressed by their dynamic mechanical properties, which can be utilized to explain the phase transitions, molecular mobility and damping properties of polymers, and the compatibility and interfacial adhesion of polymer blends [55]. The stiffness and rigidity properties of the adhesive structure were investigated through the storage modulus curve (E′) [56]. At a specific temperature, E′ initially lowers to a minimum and then increases to a maximum. The first reduction in E′ could be due to the adhesive weakening as the temperature rises. Following that, E′ begins to rise toward a maximum as the adhesive begins to gel, resulting in the formation of an unconstrained network of molecules [57]. Furthermore, a steady reduction in E′ as the temperature rises shows a high cross-link density in the glue, implying a denser network structure and greater stiffness [58]. Typically, E′ corresponds to Young’s modulus or material stiffness.

**Figure 13:**
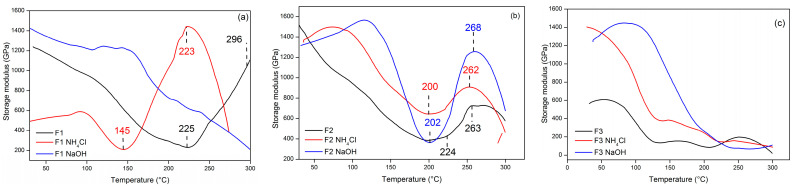
Storage modulus values of TG adhesives F1 (**a**), F2 (**b**), and F3 (**c**) using different hardeners as a function of temperature.

The reduction in the percentage of glyoxal showed a significant change in E′ (Figure 13). Adhesive formula F1 required a lower temperature to soften the adhesive, as seen from the minimum E′. Based on the type of hardener used, adhesives using NH_4_Cl hardener required lower temperatures to achieve minimum E′. In F1, the NH_4_Cl hardener reached the maximum E′ (1454 GPa) while the adhesive without hardener reached the minimum E′ (255 GPa), whereas the F2 adhesive required a high enough temperature to soften and then achieve gelation. The F3 adhesive required a temperature of more than 300 °C to soften so that in the DMA test with a testing temperature of 25 °C–300 °C, the adhesive had not softened. The high E′ value was caused by the bonds formed being tighter and stiffer so that the material can provide high resistance to the movement of the molecular chains, so the energy required will be higher to move them closer to the chain segments.

**Figure 14:**
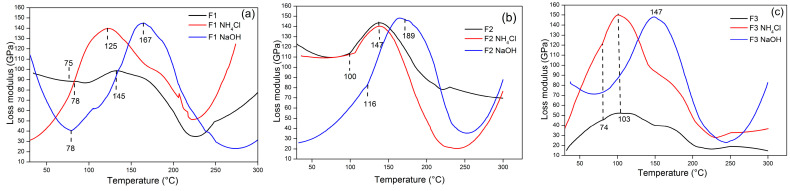
Loss modulus value of TG adhesive F1 (**a**), F2 (**b**), and F3 (**c**) with the use of different hardeners as a function of temperature.

The loss modulus (E″) trends of the TG adhesives were comparable to those of E′. The first fall in E″ was caused by the adhesive relaxing as stiffness diminished, and it subsequently continued to grow after reaching a minimum. The viscous response of a material is defined by its loss modulus. In other words, it is a measurement of the amount of energy lost as heat as a result of deformation [59,60]. Figure 14 illustrates the variation in the TG adhesive loss modulus with temperature. Figure 14a shows that the loss modulus of adhesive F1 (E″) decreased with increasing temperature and then increased significantly after 75 °C. The value of E″ in F1 without hardener at an initial temperature of 75 °C was 88 GPa and increased to 95 GPa at E″ 145 °C, while the F1 adhesive with hardeners NH_4_Cl and NaOH had an E″ value at an initial temperature of 78 °C which was 83 GPa and 41 GPa and then increased to 141 GPa and 148 GPa at E″ 125 and 167 °C. Figure 14b explains the loss modulus in adhesive F2 where adhesive with hardener NH_4_Cl and adhesive without hardener showed the same trend. The E″ of adhesives F2 and F2 NH_4_Cl reached a maximum of 158 GPa at 147 °C. Meanwhile, F2 NaOH adhesive required a higher temperature to reach maximum E″, namely 189 °C. Adhesive F3 without hardener (Figure 14c) had a much lower E″ than adhesive with hardener. This result was directly proportional to the storage modulus obtained previously. Because of the free mobility of the polymer chains, all loss modulus curves attained maximum values for mechanical energy dissipation and declined with increasing temperature. The addition of hardener resulted in increased mechanical energy dissipation.

**Figure 15:**
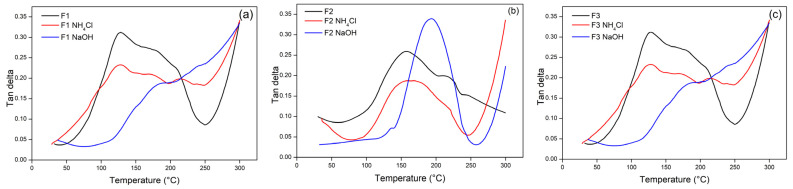
The tan delta value of TG adhesive formulas F1 (**a**), F2 (**b**), and F3 (**c**) on the use of different hardeners as a function of temperature.

The damping factor (tan δ) is defined as the ratio of loss modulus to storage modulus (tan E′/E″). The tan value is used to explore the viscoelastic features of a material, where the value (tan δ < 1) reflects the material’s elastic nature, and the value (tan δ > 1) reflects the material’s solid nature. Overall, the adhesive shows the elastic properties of the material. Figure 15 shows that tan δ increases with increasing temperature for all adhesives. In the rubber transition phase, adhesive F1 showed a lower tan delta value than the adhesive F2, and F3 exhibited a strong interlocking of molecular bonds and revealed high molecular mobility [56].The higher the tan δ value, the higher the E″ proportion and the greater the irreversible deformation capacity of the material. The tan δ value can occur due to an increase in the interaction between the hydroxyl groups formed from tannins and glyoxal which eventually incurs a polymerization reaction so that the value of the ratio of E″ to E′ will increase. The increase in the tan δ value of the adhesive was due to internal friction and viscoelastic energy dissipation, which increased with increasing temperature. This shows that a stiffer polymer molecule results in a decrease in molecular movement.

**Figure 16:**
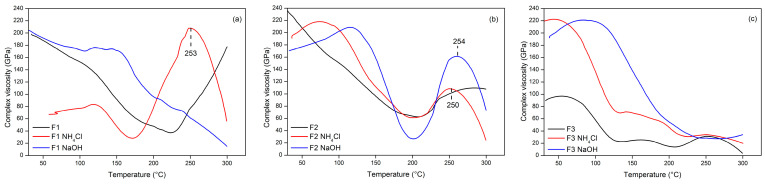
The complex viscosity of TG adhesive formulas F1 (**a**), F2 (**b**), and F3 (**c**) on the use of different hardeners as a function of temperature.

Complex viscosity (η) is the ratio of E′ to frequency and is a measure of resistance to flow. Increased complex viscosity indicates higher tensile strength and a harder tensile-fracture surface, implying better interfacial adhesion between the two components [61]. Complex viscosity has the same trend as storage modulus (Figure 16). The initial increase in the viscosity of the complex is due to the material used to place the adhesive, not the viscosity of the adhesive complex. The viscosity of the adhesive complex was detected starting at 253 °C for F1 NH_4_Cl, then the F2 adhesive tended to have the highest complex viscosity value at the same temperature of 250 °C; the F3 adhesive up to 300 °C did not show a good complex viscosity value. This indicates that the F1 adhesive had stronger interfacial adhesion properties.

### 3.4. Adhesion Properties of TG Adhesive to Areca PB

**Figure 17:**
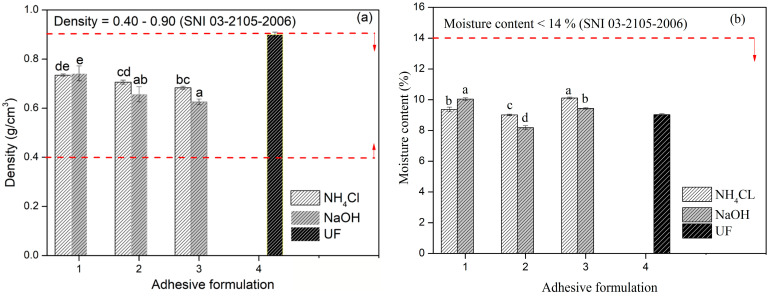
Density (**a**), and moisture content (**b**) of areca particleboard bonded with a TG adhesive at different formulations. The values different letter (a–e) are significantly different.

Figure 17a presents the effect of the TG ratio and the type of hardener on the density of the PB produced. The target density value of the PB fabricated in this work was 0.80 g/cm^3^, while the highest density value of 0.74 g/cm^3^ was obtained using the F1 adhesive. There was a decrease in the density value along with a decrease in the percentage of glyoxal in the adhesive. Even though the density of the PB decreased from F1 to F3, this density value still met the required value (SNI 03-2105-2006) for PB, namely 0.40–0.90 g/cm^3^. The difference between the target density and the results from the manufactured particleboard may have been caused by the large number of particles that were wasted during the process of making the PB. Figure 17b presents the effect of the TG ratio and the type of hardener on the MC of areca PB. The maximum allowable MC (SNI 03-2105-2006) for general purpose panels (Type 8) is <14%. The MC of the PB ranged from 8.49 to 10.22%, which is acceptable with reference to SNI 03-2105-2006, and all test panels fulfilled the level required for the intended purpose.

**Figure 18:**
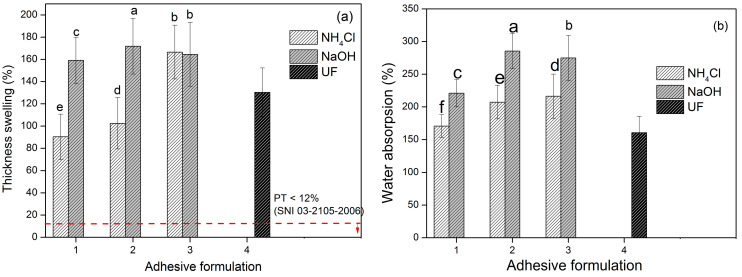
Thickness swelling (**a**), and water absorption (**b**) of areca particleboard bonded with TG adhesive at different formulations. The values different letter (a–f) are significantly different.

Thickness swelling and water absorption were calculated after 2 h of immersion (Figure 18). This PB did not meet the standard requirements of SNI 03-2105-2006, which is a maximum of 12%. The thickness swelling value and water absorption are inversely proportional to the density value. The lower the density, the thicker the expansion and the greater the water absorption. This was because there were more cavities in this sample so that more water was absorbed. The PB produced with NH_4_Cl hardener adhesive had lower thickness swelling and water absorption values compared to that with NaOH hardener. The NH_4_Cl hardener accelerated the hardening reaction when making particleboard at high temperatures so that it could produce thick expansion values and good absorption. The reaction that occurred between water and particleboard will be reconfirmed using PB hydrolysis testing. Statistical analysis revealed that the combination of adhesive formulation and catalyst significantly affected the thickness swelling and water absorption of the PB. Formula 1 combined with NH_4_Cl resulted PB panels with better thickness swelling (e) and water absorption (f), then followed by Formula 2 (d,e) and Formula 3 (b,d). Utilizing NaOH as catalyst for all adhesive formulation resulted lower thickness swelling and water absorption compared to NH_4_Cl.

**Figure 19:**
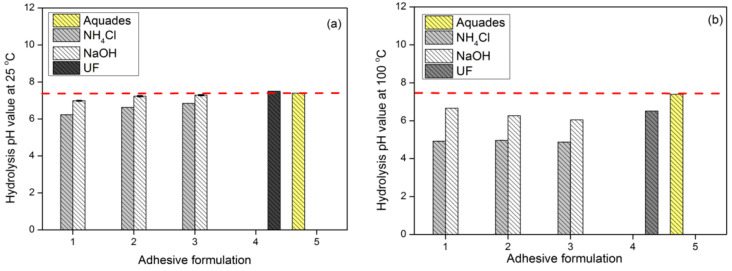
Hydrolysis pH value at 25 °C (**a**) and at 100 °C (**b**). The red dotted line is the pH of aquades.

Penetration of water molecules into the adhesive on the PB will form a chain of hydrolysis reactions. Adhesives that react with water are indicated by changes in the pH of the initial water test. The pH of the distilled water used at the beginning of the study was 7.4 and then changed after the hydrolysis test (Figure 19). This shows that there was a reaction between the adhesive, areca particles, and water, caused by a change in temperature which also caused a color change in the hydrolysis solution (Table 5 and Table 6). The particleboard produced with NH_4_Cl hardener had an acidic pH while the PB with NaOH hardener had a weak acidic pH. The change in the pH of the PB with the NaOH hardener from alkaline to acidic was due to the adhesive bonding with the acidic particles. The hydrolysis process at 100 °C caused more adhesive to come out of the board than at 25 °C.

**Figure 20:**
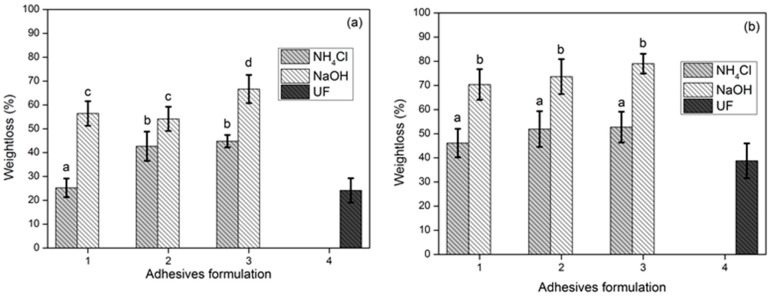
Weight loss of areca particleboard bonded with different adhesive formulations after hydrolysis at 25 °C (**a**) and at 100 °C (**b**). The values different letter (a–d) are significantly different.

This hydrolysis process caused weight loss in the PB due to the breaking of the bonds between the adhesive and the areca nut frond particles. The PB F1 with NH_4_Cl adhesive had almost the same weight loss percentage as particleboard with commercial UF adhesive in the hydrolysis process at 25 °C (Figure 20a). The results of the analysis of variance showed that the adhesive formula and the type of hardener used had a significant effect on the percentage of weight loss at 25 °C. The higher the percentage of glyoxal, the less weight loss occurs in the 25 °C hydrolysis process, while the results of the analysis of variance on weight loss during the hydrolysis process at 100 °C showed that the interaction between the adhesive formula and the type of hardener and the adhesive formula had no significant effect. Statistical analysis revealed that Formula 1 combined with NH_4_Cl resulted PB panels with lower weightloss (a), then followed by Formula 2 (b) and Formula 3 (c). Utilizing NaOH as catalyst for all adhesive formulation resulted higher weightloss compared to NH_4_Cl. It can be concluded that the use of NH_4_Cl as a hardener in the process of making areca frond PB using TG adhesives provides better particle and adhesive bonds than the use of NaOH as a hardener in the hydrolysis process, even at 100 °C (Figure 20b).

**Figure 21:**
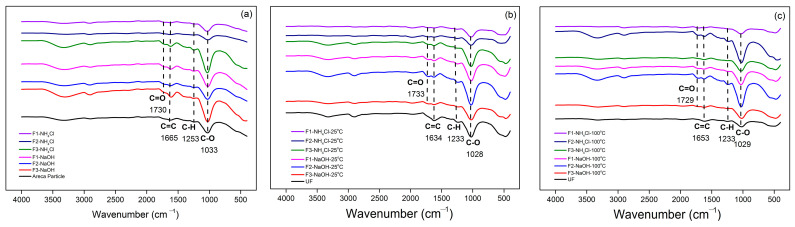
FTIR spectra of areca particleboard before hydrolysis (**a**), after hydrolysis at 25 °C (**b**), and after hydrolysis at 100 °C (**c**).

Confirmation of the functional groups of PB both before and after the hydrolysis process using FTIR yielded relatively similar absorption peaks, as shown in Figure 21. At wavenumber 2051 cm^−1^, there was absorption of the aromatic C=C functional group and then there was an absorption peak at wave number 1730 cm^−1^ which is the C=O functional group. The C=O functional group at this wave number is a functional group derived from a TG adhesive because it is not detected in the particleboard spectrum before hydrolysis (Figure 21a). In Figure 21b,c, the C=O functional group experienced a shift in the absorption peak at wave numbers 1733 cm^−1^ and 1729 cm^−1^. Based on the absorption intensity of the functional groups, the F1 NH_4_Cl adhesive did not seem to experience much difference compared to other adhesives. This is directly proportional to the pH and weight loss after hydrolysis.

**Figure 22:**
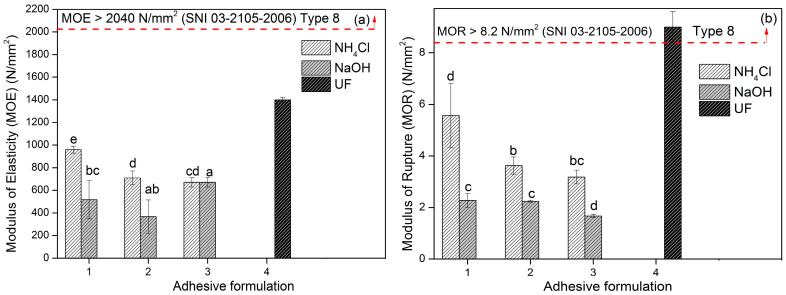
MOE (**a**), and MOR (**b**) of areca particleboard bonded with different TG adhesive formulations. The values different letter (a–d) are significantly different.

Figure 22a shows the relationship between the adhesive formula and the type of hardener and the modulus of elasticity (MOE) of PB. The MOE value showed a decreasing trend as the percentage of glyoxal decreased. This was directly proportional to the PB density [62]. The MOE value became lower when the glyoxal percentage was lowered. The results of analysis of variance showed significant differences between the types of hardener and different adhesive formulas on flexural strength. The use of NH_4_Cl hardener produced a PB that had much better bending strength than particleboard with NaOH hardener. However, this particleboard still did not meet the requirements of SNI 03-2105-2006 type 8, namely a minimum MOE value of 2040 N/mm^2^. The modulus of rupture (MOR) of PB (Figure 22b) showed the same trend as the MOE. SNI 03-2105-2006 requires a minimum MOR value of 8.2 N/mm^2^. The results of analysis of variance showed significant differences between the type of hardener and the different adhesive formulas on fracture toughness. However, the interaction between the adhesive formula and the type of hardener had no significant effect.

**Figure 23:**
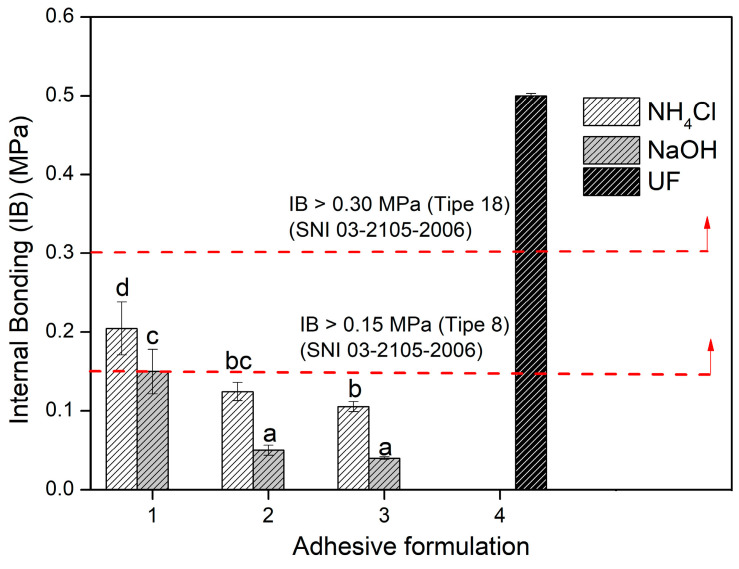
Internal bonding strength of areca particleboard bonded with different TG adhesive formulations. The values different letter (a–d) are significantly different.

The IB strength is one of the mechanical properties of PB which indicates the high value of the bonding and bonding power between the constituent materials that are combined to form PB. SNI 03-2105-2006 type 8 requires a minimum IB strength of 0.15 MPa for PB. The F1 adhesive formula used the hardener NH_4_Cl and NaOH to produce PB that meets the adhesive firmness requirements (Figure 23). The adhesive firmness decreased with decreasing glyoxal percentage in the TG adhesive ratio. The results of the analysis of variance showed significant differences between the different types of hardener and adhesive formulas. However, the interaction between the adhesive formula and the type of hardener had no significant effect.

## 4. Conclusions

The cohesion and adhesion performance of tannin-glyoxal adhesives made with different formulations and hardener types and used as areca-based particleboard binder were studied in this study. The results revealed that the yield of tannin extracted from *A. mangium* bark with hot water was 23.22%. Tannin extract shows the presence of hydroxyl groups in its structure, so it can be used as a substitute for phenol in phenol formaldehyde in the development of particleboard adhesives. The F1 tannin-glyoxal adhesive formulation (tannin: glyoxal ratio of 1:2) was characterized by a higher solids content, specific gravity, viscosity, and crystallinity than the F2 (1:1) and F3 (2:1) adhesives. The F1 adhesive required a lower temperature to achieve maximum storage modulus and loss modulus. The F1 adhesive formulation exhibited good adhesive and cohesive properties characterized by its rheological properties and viscoelastic abilities. All three adhesive formulations had a better shelf life than commercial adhesives. When served as a binding agent for areca-based PB, the performance of the tannin-glyoxal adhesive was clearly affected by the types of hardeners used. The use of NH_4_Cl as a hardener resulted in areca-based particleboards with superior properties compared with the particleboards bonded with NaOH as a hardener. Areca-nut particleboard using the TG F1 adhesive had higher MOE, MOR and IB values than those particleboards produced using the F2 and F3 adhesives.

## Figures and Tables

**Table 1:** Formulation of TG adhesive at different ratios.

Formulation (T:G)	Tannin (g)	Glyoxal (g)	Total
F1 (1:2)	40	80	120
F2 (1:1)	60	60	120
F3 (2:1)	80	40	120

**Table 2:** Adhesive formulations used for manufacturing areca-based particleboards.

Composition	F1	F2	F3
Adhesive (g)	123.08	127.42	158.81
Hardener (g)	0.74	0.76	0.95
Wax (g)	6.45	6.45	6.45
Particle (g)	644.63	644.63	644.63

**Table 3:** Characterization of tannin obtained from Acacia mangium bark.

Parameters	Value	References
Yield (%)	23.22 ± 0.95	26.43 [40]
Moisture content (%)	6.96 ± 0.37	5.47 [40]
Solids content (%)	91.36 ± 0.74	93.46 [40]
Stiasny number	73.17 ± 0.74	79.09 [41]

**Table 4:** Characterization of tannin-glyoxal adhesives developed in this work.

Formulation	Solid Content (%)	Specific Gravity	Viscosity (mPa.s)			Gelation Time (min)		
			NH	NH_4_Cl	NaOH	NH	NH_4_Cl	NaOH
F1	48.75 ± 3.27 ^b^	1.38 ± 0.01 ^c^	32.86 ± 0.09 ^i^	27.39 ± 0.11 ^h^	10.59 ± 0.12 ^e^	66.60 ± 1.34 ^g^	20.20 ± 1.15 ^b^	61.63 ± 0.75 ^f^
F2	47.09 ± 0.86 ^b^	1.35 ± 0.01 ^b^	23.77 ± 0.14 ^g^	21.35 ± 0.09 ^f^	8.54 ± 0.11 ^c^	37.16 ± 0.85 ^e^	18.67 ± 1.05 ^a^	45.23 ± 1.64 ^d^
F3	37.78 ± 0.43 ^a^	1.31 ± 0.00 ^a^	10.34 ± 0.07 ^d^	9.89 ± 0.07 ^b^	0.27 ± 0.15 ^a^	82.16 ± 1.20 ^i^	26.26 ± 0.83 ^c^	78.83 ± 0.84 ^h^

Information: NH = no hardener; NH_4_Cl = acid hardener; and NaOH = alkaline hardener; Different letters in the mean column indicate a significant difference based on Duncan’s test (α = 0.05).

**Table 5:** Color change in areca-based particleboard hydrolysis solution at 25 °C for 1 h.

Hydrolysis	F1 NH_4_Cl	F2 NH_4_Cl	F3 NH_4_Cl	F1 NaOH	F2 NaOH	F3 NaOH	UF
Before	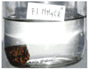	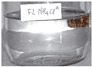	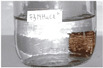	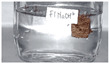	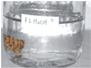	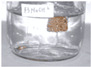	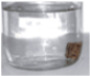
After	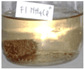	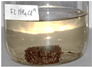	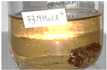	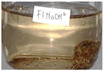	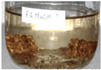	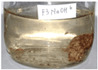	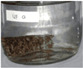

**Table 6:** Color change in areca-based particleboard hydrolysis solution at 100 °C for 1 h.

Hydrolysis	F1 NH_4_Cl	F2 NH_4_Cl	F3 NH_4_Cl	F1 NaOH	F2 NaOH	F3 NaOH	UF
Before	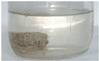	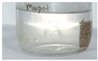	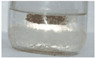	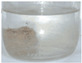	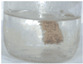	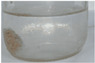	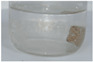
After	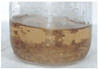	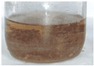	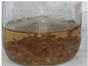	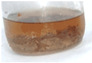	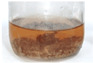	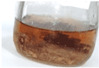	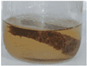

## Data Availability

The data presented in this study are available on request from the corresponding authors.
